# Guillain–Barre syndrome associated with hepatitis E virus infection: A case report

**DOI:** 10.1002/ccr3.7863

**Published:** 2023-08-29

**Authors:** Ayman Ahmed, Sarah Misbah EL‐Sadig, Emmanuel Edwar Siddig

**Affiliations:** ^1^ Institute of Endemic Disease University of Khartoum Khartoum Sudan; ^2^ Swiss Tropical and Public Health Institute (Swiss TPH) Allschwil Switzerland; ^3^ University of Basel Basel Switzerland; ^4^ Faculty of Medicine University of Khartoum Khartoum Sudan; ^5^ Faculty of Medical Laboratory Sciences University of Khartoum Khartoum Sudan

**Keywords:** critical care medicine, infectious disease, neurology, transdisciplinary one health strategy

## Abstract

**Key Clinical Message:**

Hepatitis E virus (HEV) infection can be manifested with several neurological syndromes including GBS. Therefore, healthcare providers should consider HEV in their differential diagnosis for patients with neurological disorders.

**Abstract:**

We report a case of Guillain‐Barré syndrome associated with hepatitis E virus infection. The current case‐report demonstrates diagnostic challenge to identify GBS case in a limited‐resources country like Sudan. However, HEV infection should be highly suspected in patients with neurological manifestation with high liver enzymes.

## Graphical abstract

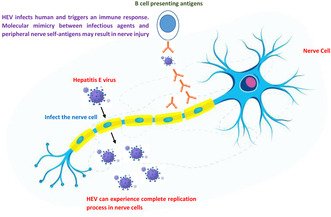

## 1 INTRODUCTION

Guillain‐Barré Syndrome (GBS) is an acute inflammatory disease that affects the peripheral nervous system. It is caused by an aberrant immune response triggered by viral, bacterial, or parasitic infections, a phenomenon known as molecular mimicry.^1^ Several infectious agents have been confirmed to be involved in the development of GBS, including but not limited to COVID‐19, hepatitis E and B viruses, malaria, Influenza A virus, as well as major arboviruses like dengue, West Nile, and Zika viruses.^1^, ^2^, ^3^ These infections can lead to an immune response that mistakenly targets the peripheral nerves, causing the characteristic demyelination and dysfunction of both motor and sensory nerve fibers observed in GBS. Interestingly, it has been observed that approximately two thirds of patients with GBS experience respiratory or gastrointestinal symptoms before the onset of the disease. Surprisingly, a third of the patients remain asymptomatic until the clinical presentation of GBS.^4^ This fact emphasizes the challenges faced in the surveillance and early detection of GBS. Consequently, the global, regional, and national burden of GBS is significantly underestimated, especially in resource‐limited settings such as Sudan.^1^

Hepatitis E virus (HEV) infection is one of the most common causes of acute viral hepatitis and acute jaundice syndrome worldwide. HEV infection is an important global health issue that contribute significantly in the global morbidity and mortality due to infectious diseases.^5^, ^6^ The World Health Organization (WHO) estimates that annually over 20 million cases and around 45,000 deaths related to HEV infection reported worldwide.^6^ The clinical presentation of HEV infection could be classified into hepatic and extra‐hepatic form of the disease, with the hepatic form further subdivided into acute hepatitis, fibrosis, or cirrhosis.^7^ The extra hepatic manifestation of HEV includes neurological, hematological, and renal disorders. Interestingly, 16% of acute hepatitis E infection involves neurological manifestations.^8^ High proportion of HEV infection is associated with neuralgic amyotrophy and in GBS.^9^ HEV infection was confirmed in 5%–11% of patients presented to outpatient clinics in Netherlands, Japan, Belgium, and Bangladesh.^10^, ^11^, ^12^, ^13^, ^14^

In Sudan, the prevalence of HEV is rapidly growing during the recent years; this rapid growth is mainly influenced by climate changes.^15^ In this communication, we report the first case of Guillain–Barre syndrome associated with HEV infection from Sudan.

## 2 CASE PRESENTATION

A 33‐year‐old male presented to the Omdurman Teaching Hospital with fatigue, cough, mild jaundice, and he reported having dark color urine for 9 days. The patient was referred to the general hospital Royal Care in Khartoum state where a blood sample was collected. The liver function test showed an elevated level of aspartate aminotranferease (AST) 290 U/L, alanine aminotransferease (ALT) of 466 U/L, and total bilirubin of 3.3 mg/dL, direct bilirubin of 1.3 mg/dL. Based on the clinical presentation, the patient was tested for hepatotrophic viruses including adenovirus, hepatitis A, B, C, D, and E viruses using an enzyme‐linked immunosorbent assay (ELISA); all were negative except for HEV infection that came positive for HEV‐IgM. Additionally, the patient was screened for various related infections that manifest the liver including yellow fever, malaria, and Rift Valley fever and all were negative.

After 8 days, the patient arrived at the emergency room (ER) complaining of distal ascending paraesthesia to the elbow and knee, and he was unable to walk without assistance. The patient reported that 24 h prior to admission, he experienced lower limb weakness, numbness, and tingling sensation in hands and feet. Neurological examinations revealed mild lower limb paralysis (Medical Research Council scale (MRC) 5/5 at upper limbs and 1/5 at lower limbs), mild hypoesthesia (reduced sensation to pinprick distal to the ankle joint), and generalized areflexia with down going plantar reflex. He reported no history of blood transfusions, risky sexual behavior, or sharing needles.

**FIGURE 1:**
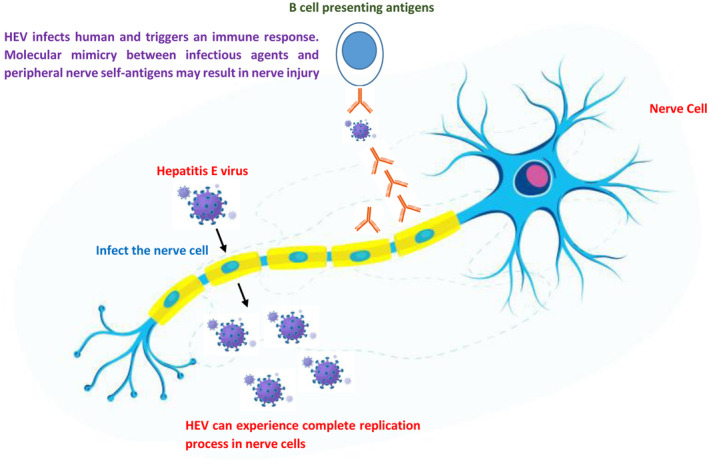
Illustrates the likely pathways through which hepatitis E virus may trigger the development of Guillain‐Barré Syndrome.

Therefore, the involvement of GBS was suspected, and lumbar puncture was conducted on the second day of admission to the hospital. Cerebrospinal fluid (CSF) examination showed 0/μL monocyte, random blood glucose was 4.6 mmol/L, and protein level was 275.3 mg/dL, which suggested albuminocytologic dissociation. Nerve conduction investigations showed evidence of demyelinating neuropathy with dysfunction of motor and sensory nerve fibers. This confirmed the involvement of GBS due to HEV infection. Figure 1 illustrate the mechanism of HEV‐induced GBS development (Figure 1).

The patient was treated with intravenous immunoglobulin at a dose of 0.4 mg/kg per day for 5 days. During the next 2 weeks, his clinical condition and muscle power improved gradually, and the patient had no complaints of respiratory distress or malaise. Repeat CSF examination 2 weeks after admission revealed 10/μL monocyte and 85.7 mg/dL protein level.

## 3 DISCUSSION

Hepatitis E is a water borne and enterically transmitted infection that is hyper‐endemic in low middle‐income countries and mesoendemic in developed countries.^15^, ^16^ The virus is well recognized to induce hepatitis; however, neurological syndromes associated with HEV infection cause significant morbidity.^17^ Limited data exists regarding the neurological consequences of HEV infection, with most studies originating from India and involving HEV genotype 1.^18^ However, several developed countries have recently reported an uptick in HEV infections (primarily genotype 3) resulting in GBS.^18^ A study conducted in the Netherlands found that 10 GBS patients had a higher concentration of anti‐HEV immunoglobulin (IgM) antibodies (5.0%) compared to a healthy control (0.5%), resulting in an odds ratio of 10.5, and a 95% confidence interval of 1.3–82.6. HEV RNA was detected in the blood of three of these patients.^11^

Neurological complications of HEV infection reported so far includes; GBS and neuralgic amyotrophy considered as the most commonly associated conditions. Interestingly, the virus can also lead to transverse myelitis, encephalitis, cranial nerve palsy, and meningoradiculitis.^11^, ^17^

GBS is the most common cause of acute flaccid paralysis, however, it is challenging to diagnose in limited capacity and resources settings as it requires a complete medical history, neurological examination, electrophysiological test, and CSF analysis.^19^ In addition, healthcare providers need to understand that electromyography (EMG) results are typically normal during the acute phase. Therefore, it is crucial and mandatory to gather a comprehensive medical history and conduct thorough clinical examinations.

Several hypotheses were developed about mechanisms of HEV inducing GBS, however up to date, the exact mechanism is still unclear.^20^ The possible scenarios according to the published literature includes either by the direct viral damage due to the replication of the virus within the nervous system or by indirect way that is, immune response or what is called as molecular mimicry.^21^, ^22^, ^23^ In Figure 1, we illustrate mechanisms of developing GBS due to HEV infection (Figure 1).

Our case report, urges for further investigation to estimate the actual burden of hepatitis E and the burden of the associated GBS. Particularly in countries like Sudan, where heavy burden of wide range of infectious diseases with overlapping symptoms and potential involvement in GBS development including Covid‐19, malaria, and several arboviruses are prevalent in the country.^1^, ^24^, ^25^ Major arboviral diseases that are associated with GBS and endemic in the country include Chikungunya, Crimean‐Congo Hemorrhagic fever (CCHF),^26^ dengue, Yellow fever, and Rift Valley fever.^24^, ^27^ Therefore, reducing the burden and prevalence of GBS in Sudan and similar settings would require the implementation of a transdisciplinary One Health strategy with an integrated surveillance and response system for the early preparedness, detection, and response.

In summary, GBS is an extrahepatic manifestation of HEV infection. Furthermore, HEV infection should be suspected in patients with neurological manifestations with increased liver enzymes. In this case, CSF examination demonstrated an elevated level of proteins with pleocytosis, which supported our suspicion of GBS involvement. Healthcare providers should be attention to such association of clinical presentations to improve and facilitating making the accurate diagnosis as early as possible for a better case management.

## AUTHOR CONTRIBUTIONS

**Ayman Ahmed:** Conceptualization; data curation; formal analysis; investigation; methodology; resources; supervision; validation; visualization; writing – original draft; writing – review and editing. **Sarah Misbah EL‐Sadig:** Conceptualization; data curation; formal analysis; investigation; methodology; writing – review and editing. **Emmanuel Edwar Siddig:** Conceptualization; data curation; formal analysis; investigation; methodology; resources; supervision; validation; visualization; writing – original draft; writing – review and editing.

## FUNDING INFORMATION

None.

## CONFLICT OF INTEREST STATEMENT

The author reports no conflicts of interest in this work.

## CONSENT

Written informed consent was obtained from the patient to publish this report in accordance with the journal's patient consent policy.

## Data Availability

The data that support the findings of this study are available from the corresponding author upon reasonable request.
